# Correlations between autism spectrum disorders and childhood apraxia of speech

**DOI:** 10.1192/j.eurpsy.2021.557

**Published:** 2021-08-13

**Authors:** C. Cabral, F. Fernandes

**Affiliations:** 1 Fonoaudiologia, Fisioterapia E Terapia Ocupacional, Universidade de Sao Paulo, São Paulo, Brazil; 2 School Of Medicine, universidade de São Paulo, São Paulo, Brazil

**Keywords:** autism, apraxia, Children, language

## Abstract

**Introduction:**

Autism Spectrum Disorder (ASD), is a neurodevelopmental disorder, characterized by inabilities in communication and social interaction. ¹ Childhood Apraxia of Speech (CAS) is a neurological disorder in which the consistency and precision of speech movements are impaired, in the absence of neuromuscular deficits.^2^ Research indicates that children with ASD do not have a higher prevalence of CAS.^3^ It is suggested that comorbid ASD and CAS would be expected to be extremely rare.

**Objectives:**

Verify the occurrence of CAS in children with ASD.

**Methods:**

The study included 22 children diagnosed with ASD aged between 4 and 8 years, who were undergoing speech therapy at a specialized health service and their therapists. The test was applied by the therapists Differential Assessment of Autism and Other Developmental Disorders (DAADD)^4^, divides into six areas of development: language, pragmatic, sensory, motor, physical and behavioral to differentiate and diagnose disorders of neurological origin.

**Results:**

Among the 22 children participating in the research, 20 did not score the item apraxia. Only two children were referred with apraxia and twelve had receptive language and pre-academic skills proportional to their age. Of 22 participants, only three were overly excited for verbal productions.

**Conclusions:**

The analyzes of data suggests that the occurrence of CAS in children with ASD is low and underlying the disorder.

